# In Vitro Differentiation of Embryonic and Adult Stem Cells into Hepatocytes: State of the Art

**DOI:** 10.1634/stemcells.2008-0963

**Published:** 2009-03

**Authors:** Sarah Snykers, Joery De Kock, Vera Rogiers, Tamara Vanhaecke

**Affiliations:** Department of Toxicology, Vrije Universiteit BrusselBrussels, Belgium

**Keywords:** Adult stem cells, Embryonic stem cells, Hepatocytes, In vitro protocols, Differentiation

## Abstract

Stem cells are a unique source of self-renewing cells within the human body. Before the end of the last millennium, adult stem cells, in contrast to their embryonic counterparts, were considered to be lineage-restricted cells or incapable of crossing lineage boundaries. However, the unique breakthrough of muscle and liver regeneration by adult bone marrow stem cells at the end of the 1990s ended this long-standing paradigm. Since then, the number of articles reporting the existence of multipotent stem cells in skin, neuronal tissue, adipose tissue, and bone marrow has escalated, giving rise, both in vivo and in vitro, to cell types other than their tissue of origin. The phenomenon of fate reprogrammation and phenotypic diversification remains, though, an enigmatic and rare process. Understanding how to control both proliferation and differentiation of stem cells and their progeny is a challenge in many fields, going from preclinical drug discovery and development to clinical therapy. In this review, we focus on current strategies to differentiate embryonic, mesenchymal(-like), and liver stem/progenitor cells into hepatocytes in vitro. Special attention is paid to intracellular and extracellular signaling, genetic modification, and cell-cell and cell-matrix interactions. In addition, some recommendations are proposed to standardize, optimize, and enrich the in vitro production of hepatocyte-like cells out of stem/progenitor cells.

## INTRODUCTION: THE STEM CELL MICROENVIRONMENT

**Figure 1:**
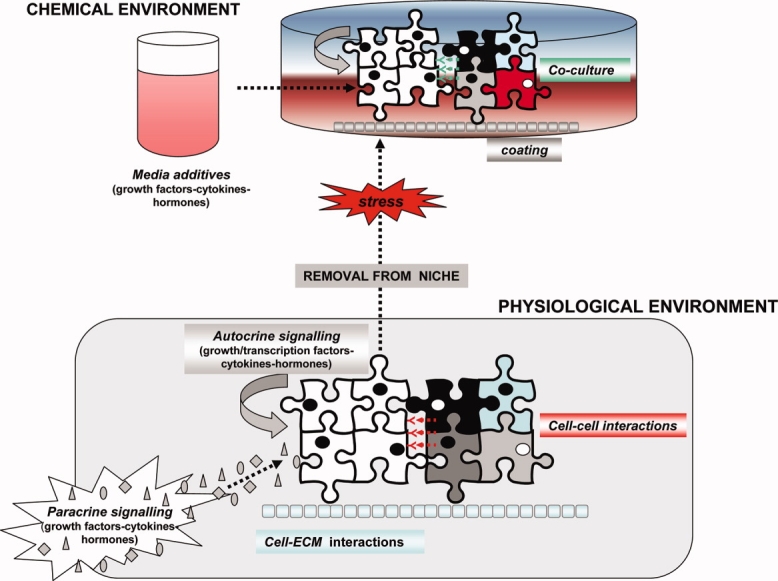
Adult stem/progenitor cell environment in vivo and ex vivo. The balance between cell growth/differentiation of adult stem/progenitor cells is regulated by a complex cross-talking network of paracrine and autocrine signals and cell-cell and cell-extracellular matrix interactions. Abbreviation: ECM, extracellular matrix.

The totipotent fertilized egg is the ultimate stem cell that gives rise to all tissues of the developing embryo. In the adult, “multipotent” stem/progenitor cells reside for a nearly infinite term at restricted locations to allow continuation of the cycle of life [1–3]. These so-called stem cell niches have been identified in the bone marrow [4], brain [5], skin [6], intestinal crypt [7], and liver [1, 8]. The original idea of a stem cell “niche” evolved from the concept that stem/progenitor cells inhabit tissues within an “inductive microenvironment” that directs their self-renewal, differentiation, and cell fate in both normal physiology and disease [1, 3, 9]. Many developmental regulatory signaling molecules, including Wnts, bone morphogenic proteins (BMP), fibroblast growth factors (FGFs), Notch, and others, may play a role [1, 7, 8]. In addition to stem/progenitor cells, the niche microenvironment comprises nonstem niche cells (e.g., stromal cells, periductular fibroblasts, and stellate cells), parasympathetic nerve endings and specialized extracellular matrix (Fig. 1) [1, 2, 10, 11]. Other cell-cell interactions have also been hypothesized. The coordinated signaling between component cells and scaffold, (in)direct cell-cell contacts, and integration of stem cell-autonomous properties represent an interactive and dynamic system, organized to facilitate cell fate decisions in a proper spatiotemporal manner [1, 2, 8].

Historically, the developmental paradigm was that adult stem cells were, in contrast to their embryonic counterparts, subjected to “cell fate determinism.” Nowadays, new insights on stem cell potency have challenged the latter canonical developmental hierarchy [12]. Nevertheless, “adult stem cell plasticity” still remains an obscure and rather rare phenomenon. The finding that at least some transitions may be ascribed to cellular fusion events have underpinned true plastic phenomena [13, 14] and has led to an outbreak of raw headlines, utterly questioning adult stem cell versatility, for example, “Adult Stem Cell Plasticity—Fact or Artifact?” [15], “Recipes for Adult Stem Cell Plasticity: Fusion Cuisine or Readymade?” [16], “Adult Stem Cell Plasticity—Fact or Fiction” [17], and “Stem Cell Fusion Confusion” [18]. Hitherto, the answer remained an open question. The fact is that not all cellular “redirections,” no matter how rare their occurrence, might be ascribed to simple fusion events [12, 16, 19, 20]. In vitro, spontaneous fusion only occurs in coculture models, and, even so, the frequency rate is limited to about one fusion event per 10^3^–10^6^ cocultured cells [12, 20, 21]. In addition, in vivo, regular natural fusion of stem cells with other cell types seems unlikely because, with the exception of the liver and the pancreas, healthy organs lack substantial complements of polyploid cells [16]. This brings us back to the essence of fate reprogrammation of stem/progenitor cells: the stem cell microenvironment. In vivo, an injured environment seems most favorable for tissue replenishment by stem/progenitor cells [16, 22, 23], although extracellular cues provided by the transplanted stem/progenitor cells (cf. the “bystander” effect) also may be partly accountable for “recovery” of the recipient [23, 24]. In vitro, the highest success rates of phenotypic “diversification” were gained upon mimicking the microenvironment (Fig. 1). It is now well recognized that identification of the in vivo signaling patterns —the lineage-specific growth factors/cytokines and their (relative) dose and rank of application [8]—is crucial for eliciting distinct responses from cultured stem/progenitor cells and directing lineage-specific cell growth and differentiation in vitro. Apart from the latter cues, intrinsic cellular stress signals, executed by removal of stem/progenitor cells from their physiological niche, may also facilitate alterations in cellular architecture and phenotype via mechanisms of “cytoskeleton collapse” (Fig. 1; see also Need for Standardization, Optimization, and Enrichment) [25, 26].

In this survey, we provide an up-to-date overview on the wide variety of experimental conditions that have been applied thus far to trigger cultured pluripotent embryonic stem (ES) cells, multipotent mesenchymal(-like) stem/progenitor cells (MSCs), and bipotent liver progenitor cells (LPCs) into (functional) hepatocytes (Tables 1, 2,, and 3). In principle, most approaches are based on reconstructing the in vivo microenvironment via (a) addition of soluble medium factors and (b) reconstitution of cell-matrix, and (c) cell-cell interactions. Recently, (d) interest has also increased in chromatin modulation as a strategy to manipulate cell fate. Constitutive overexpression of liver-enriched transcription factor (LETF) genes might be an alternative but has a downside too.

**Table 1:** Strategies for in vitro differentiation of ES cells into hepatocyte-like cells including their molecular and functional endpoints

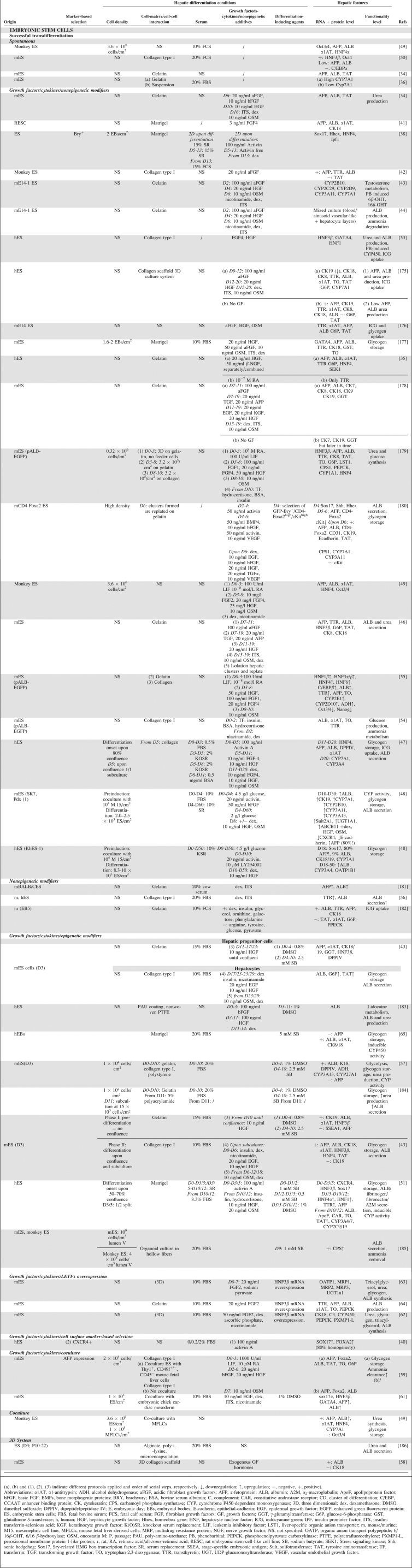

**Table 2:** Strategies for in vitro differentiation of MSCs into hepatocyte-like cells including their molecular and functional endpoints

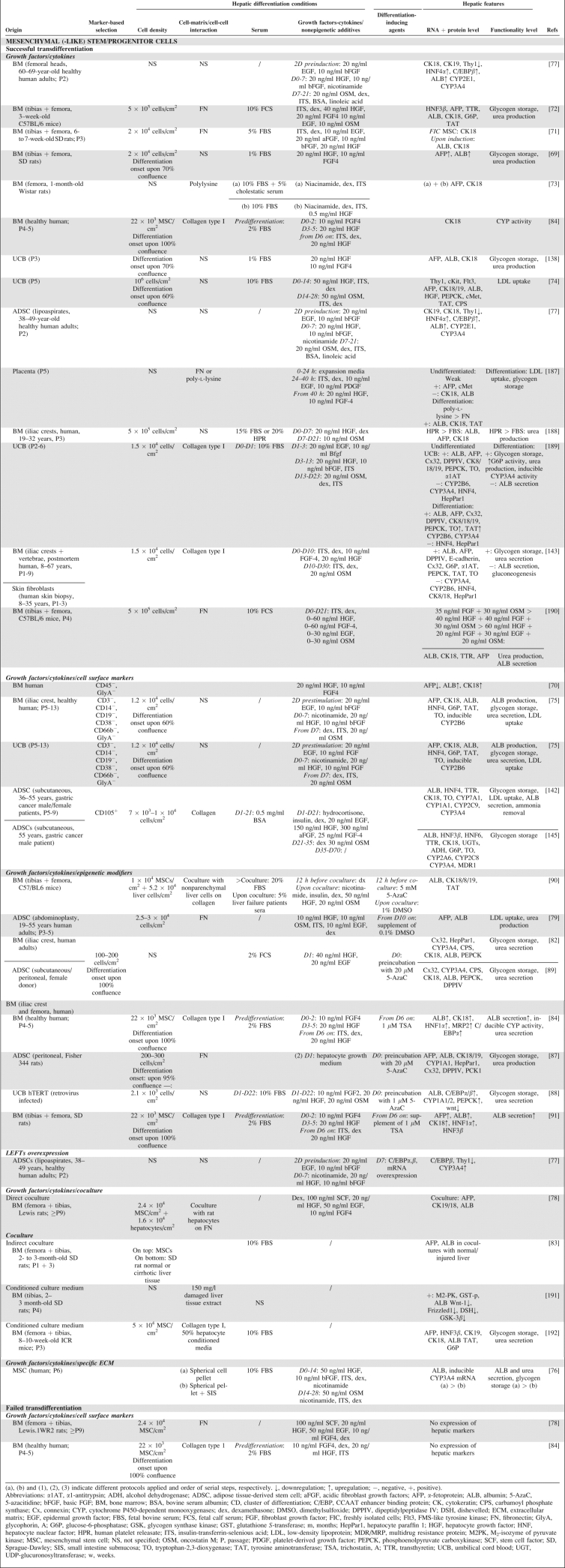

**Table 3:** Strategies for in vitro differentiation of LPCs into hepatocyte-like cells including their molecular and functional endpoints.

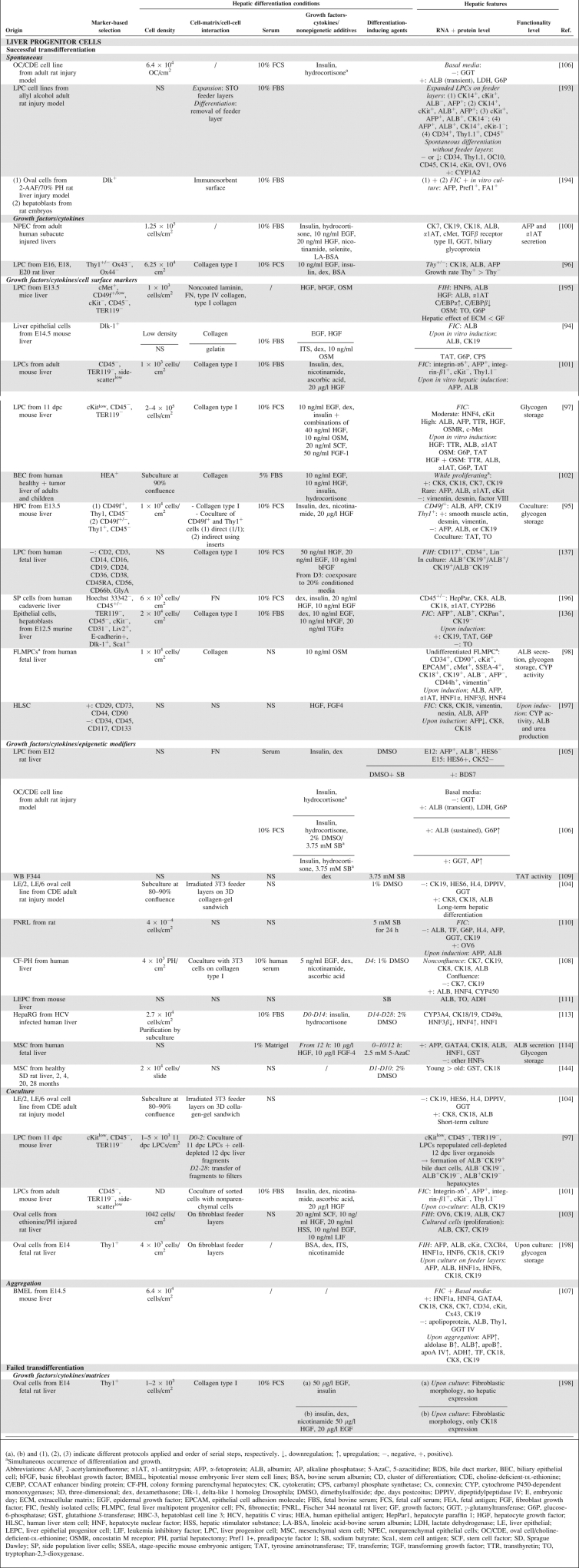

## FROM STEM CELLS TO HEPATOCYTES: HEPATOGENESIS IN VIVO

**Figure 2:**
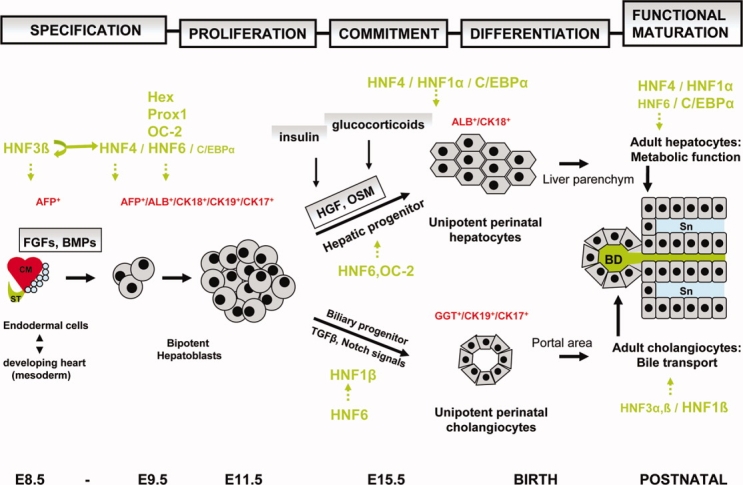
Schematic presentation of fetal liver development (modified from [2]). The establishment of a fully functional liver architecture is not accomplished before postnatal stages and follows upon a sequential array of tightly regulated intra- and extracellular signaling pathways, including liver-enriched transcription factors (LETFs) and growth factors, cytokines, glucocorticoids and hormones, respectively. To distinguish the level of expression and/or regulating role among diverse LETFs, different letter sizes are used. Abbreviations: ALB, albumin; AFP, α-fetoprotein, BMP, bone morphogenic proteins; C/EBP, CCAAT enhancer binding protein; CK, cytokeratin; CM, cardiogenic mesoderm; E, embryonic day in rodent liver development; FGF, fibroblast growth factors; GGT, γ-glutamyltransferase; HGF, hepatocyte growth factor; HNF, hepatocyte nuclear factor; OC-2, Onecut transcription factor; ST, septum transversum; TGF, transforming growth factor.

The microenvironment of developing hepatocytes is a continuously changing process of successively occurring biological events [27]. Each step of cell growth and differentiation is tightly regulated by intra- and extracellular communication, as well as cell autonomous mechanisms (Fig. 2). Nodal (activin), FGFs, BMP, hepatocyte growth factor (HGF), and oncostatin M (OSM) are herein the most essential extracellular signals [2, 27–30]. At the intracellular level, the liver-enriched transcription factors hepatocyte nuclear factor (HNF) 3α,β, HNF4α, HNF1α,β, HNF6, and CCAAT enhancer binding protein (C/EBP) α,β act consecutively, in essence, in a cross-regulatory manner, at specific developmental stages to regulate liver-specific gene expression [27–29, 31, 32] (Fig. 2).

In brief, at the onset of liver ontogeny (approximately rodent embryonic day [E] 8.5), specification from endodermal stem cells toward the hepatic epithelial lineages requires, next to HNF3β and activin A signaling, signaling from two adjacent mesodermal cell types: FGFs (FGF1 and basic bFGF) from the cardiogenic mesoderm cells, and BMPs (BMP2, BMP4, BMP5, and BMP7) from the septum transversum mesenchyme [27–29] (Fig. 2). Then (approximately rodent E9.0-9.5), cells start to massively proliferate and bud into the stromal environment of the septum transversum mesenchyme. The hepatic epithelial specified cells are now referred to as bipotent hepatoblasts (GATA4^+^, HNF4α^+^, HNF6^+^, hepatic α-fetoprotein [AFP]^+^/albumin [ALB]^+^, and biliary cytokeratin [CK 17^+^/CK19^+^) [2, 27–29]. At rodent E11-12, the liver primarily becomes a primarily hematopoietic organ. Hematopoietic stem cells (HSCs) originating from the extrahepatic organ colonize the liver bud, thereby emitting a growth signal for the liver [28, 29]. Consequently, hepatoblasts continue to proliferate and start expressing placental alkaline phosphatase, intermediate filament proteins (CK14, CK8, and CK18), γ-glutamyltransferase, and later also α_1_-antitrypsin, glutathione *S*-transferase P, C/EBPα, lactate dehydrogenase, and muscle pyruvate kinase [2, 28, 29]. As commitment progresses, three distinct cell populations are distinguished: (a) hepatocyte-committed cells that exclusively express hepatocyte markers, such as AFP and ALB, (b) cholangiocyte-committed progenitor cells, expressing biliary cell markers such as CK19, and (c) a bipotential hepatoblast population, expressing both hepatic and biliary markers. The latter population develops into biliary or hepatic cell lines and is therefore considered to be the fetal source of hepatic progenitor cells [2, 27, 29]. Differentiation along the cholangiocytic lineage is promoted by Notch signaling pathways [27, 29, 30]. HGF, excreted by mesenchymal cells or nonparenchymal liver cells, antagonizes the latter process, resulting in support of growth and differentiation of the fetal hepatocytes. The hormone insulin synergistically promotes this effect [2, 28, 33]. Subsequently, cooperative action of OSM, mostly produced by HSC, and glucocorticoids induces partial hepatic maturation and suppression of embryonic hematopoiesis [2, 27, 28, 33] (Fig. 2). OSM alone fails to induce differentiated liver phenotypes, implying the essence of glucocorticoids as triggers for hepatic maturation [28]. Upon progression of the differentiation stage, the gene expression profile of fetal liver epithelial cells acquires a more mature phenotype. In parallel, the percentage of bipotent cells is markedly reduced. At this point, although cells continue to proliferate, most of them are unipotent and irreversibly committed to either the hepatocytic or cholangiocytic lineage [2, 27, 29]. Complete functional hepatic maturation ultimately takes place after birth upon coassistance of HGF, produced by the surrounding nonparenchymal liver cells (sinusoidal, stellate, and endothelial cells) [33].

Key signaling and molecular cross-talk events are thus patterned to occur in the right place at the right time [8]. Interactions between these various compartments accomplish homeostatic regulation of stem/progenitor cell functioning in vivo [2, 8]. Consequently, identification and simulation of these in vivo signaling patterns might comprise an approach to contribute to fate reprogrammation of stem/progenitor cells in vitro.

## FROM STEM CELLS TO HEPATOCYTES: CURRENT IN VITRO DIFFERENTIATION STRATEGIES

### Embryonic Stem Cells

ES cells spontaneously differentiate into cell types of the three germ layers, including hepatocytes, upon removal of leukemia inhibitory factor and feeder layers [34–37]. The processes of lineage establishment in developing embryoid bodies (EBs) appear to follow the events of embryogenesis, suggesting that ES cells can recognize and respond to the signals regulating embryonic development. The drawback is the yield of mixed cell types.

#### Addition of Soluble Medium Factors (Growth Factors/Cytokines/Corticosteroids/Hormones)

The use of growth factors and cytokines is pivotal for hepatic growth of ES cells in vitro. Hormones and corticosteroids have a supporting role (Table 1).

Basically, activin A enriches ES cell cultures for endodermal populations [38, 39] and definite endoderm [40]. FGFs, but not BMP, are effective in mediating early hepatic differentiation, yet the choice of the most suitable FGF type depends on the species involved [41, 42]. HGF supports a midlate hepatic phenotype (e.g., ALB, dipeptidyl peptidase IV expression) [37, 43], but fails to induce functional maturation [34, 43]. Stepwise addition of FGF, HGF, and a mixture of insulin-transferrin-sodium selenite (ITS), dexamethasone, and OSM, on the other hand, seems successful [44–46]. Fine-tuning of the latter sequential strategy might even result in 70%–80% purity of ES cell-derived hepatocytes/hepatic progenitors within the culture system [47, 48]. Inherent to most differentiation protocols is the coexposure to serum. It contains hormones, growth factors, and other undefined substances that might contribute to stochastic differentiation of pluripotent ES cells [49, 50]. Nowadays, however, many efforts are being made to work under serum-free conditions. In particular, the use of serum-replacement factors has become a promising trend [47, 48, 51].

#### Reconstruction of In Vivo Cell-Matrix and Cell-Cell Interactions

Imitation of the ontogenic scaffold (particularly collagen) [42, 50, 52–58] and coculture with hepatic and nonhepatic cell types might provide an optimal in vitro environment to promote hepatogenic differentiation in ES cell and other stem cell cultures [49, 59]. However, intimate physical cell contact may result in cell fusion and requires highly sophisticated techniques to separate distinct cell populations. Hence, differentiation protocols currently use semipermeable membranes or filtered cell-conditioned media [60]. Fetal liver cells probably represent the most suitable cultivation partners [49, 59], because they, unlike cardiac mesoderm [61], contribute to hepatocellular functionality in ES cell cultures.

#### Determination of Cell Fate via Genetic Modification

(a) LETFS overexpression. HNF3β functions as a vital regulator of the initial intracellular signaling pathways in liver development/regeneration [28, 32] (Fig. 2). In addition, it may act as a driving force of ES cell differentiation along the hepatic lineage. In this context, HNF3β-transfected ES cells acquire a hepatic phenotype, more efficiently and far earlier than their untransfected counterparts [62–64]. Using this approach, ES cell differentiation in culture is in fact driven by the same transcriptional events as seen in early liver organogenesis in vivo. Hepatic gene expression and also hepatocellular functionality are found to be directly related to HNF3β levels [62]. A stated alternative is the recombinant expression of E-cadherin, most likely because adherens junction-mediated intercellular coupling has an integral role in hepatocyte functioning [52]. Major drawbacks of the constitutive overexpression of regulatory (transcription) factors are the risks of both unpredictable and nonintended gene upregulation in vitro.

(b) Epigenetic modification. The actual idea of changing cell fate via direct interference with the local chromatin structure of plastic cells was introduced only a few years ago. In 2003, ES exposure to 5 mM sodium butyrate led to 10%–15% enrichment with pure hepatic cells [65]. Lately, priming with alternating concentrations of sodium butyrate (0.5–1 mM) in the presence of activin A resulted in 10%-70% enrichment [51]. Basically, combined application of epigenetic modification and stepwise exposure to cytokine stimuli contributed considerably to homogeneity of the end population and acquirement of hepatic functionality [51]. Hitherto, both successful and failed differentiations were obtained using histone deacetylase inhibitors (HDACis), rendering their hepatotrophic effect ambiguous [43, 57]. Plausible explanations are described in From Stem Cells to Hepatocytes: Current Characterization Strategies and Their Limitations.

### Multipotent Mesenchymal Stem Cells

Unidirectional/downstream differentiation into other mesenchymal cell types, such as adipocytes, chondrocytes, and osteoblasts readily occurs in the presence of a simple cocktail of growth factors and nutrients [66]. Successful bypassing of lineage borders depends mainly on multistep processes in a coordinated, synergistic signaling network (Table 2).

#### Addition of Soluble Medium Factors (Growth Factors/Cytokines/Corticosteroids/Hormones)

Multipotent adult progenitor cells (MAPCs), discovered by Verfaillie and coworkers, were the first plastic cells found within adult bone marrow that gained the ability to undergo hepatic differentiation. Using combined exposure to FGF + HGF + ITS + dexamethasone, MAPCs transformed into cells with morphological, phenotypic, and functional characteristics of hepatocytes [67]. Yet, the resultant population was far from homogeneous. Significantly optimized differentiation was obtained via exposure of bone marrow stem cells to the same hepatogenic factors, but in a time-specific sequential manner, reflecting their secretion pattern during the hepatogenesis in vivo. More than 85% of the thus sequentially cultured cells featured a highly differentiated hepatic phenotype and functionality, including inducible cytochrome P450 (CYP)-dependent activity [68]. Thus far, several research groups have revealed expression of distinct hepatocyte markers and functions, that is, ALB and urea secretion, glycogen storage, and low-density lipoprotein uptake upon stimulation of MSCs with hepatogenic factors exclusively, added either as a mixture (FGF + HGF [69–71]; FGF + HGF + OSM [72]) or separately (HGF [73]; HGF/OSM [74]; FGF/HGF/OSM [75]). Also combinations thereof (FGF + HGF followed by OSM [76, 77]) have been applied. In contrast, others emphasized the necessity of supplementary differentiation-inducing factors to enforce functional hepatic conversion of MSCs [78]. Basically, soluble medium factors such as dexamethasone, ITS, and nicotinamide synergistically affect the hepatic driving pathways [79]. In sharp contrast to the critical role of serum in MSC expansion and until recently in ES cell differentiation, serum-free conditions have been successfully applied on a routine basis for hepatic differentiation of MSCs [75, 77–79].

#### Reconstruction of In Vivo Cell-Matrix and Cell-Cell Interactions

Cocultures of stromal bone marrow cells with primary hepatocytes were at first designated to develop long-term functional hepatic in vitro models [80]. Jagged1 protein was considered responsible for the benign effects on hepatocyte differentiation by mediating differentiation events via the Notch signaling pathway [80, 81]. Later, Jagged1 and Notch were considered essential in driving bone marrow progenitors toward hepatocyte-lineage cells [81]. In a recent study by Lange et al. [78], coculture with liver cells was claimed to be the sole trigger able to shift MSC into cells with a hepatobiliary phenotype. The impaired differentiation capability of the chosen clonal MSCs or the high purity of high passaged MSCs (thus not contaminated with hematopoietic stem cells) [82, 83] was held responsible for failing growth factor-stimulated hepatic differentiation.

Another critical factor affecting cellular differentiation status is the spatial distribution between cells. Differentiation is usually initiated upon 60%–100% confluence (Table 1). Significantly promoted hepatic differentiation in areas of highest cellular density (maximal cell-cell contact) versus that in lower cellular density [67, 68, 74, 76, 82, 84] emphasized the relevance of intercellular communication during differentiation processes. Minor roles are ascribed to the type of coatings used. The natural scaffold collagen turns out to be most effective [68, 84].

#### Determination of Cell Fate via Genetic Modification

(a) LETFS overexpression. To the best of our knowledge, only one study thus far has investigated the putative inductive effect of LETFs on hepatic differentiating MSCs. More specifically, Talens-Visconti et al. [77] confirmed the contribution of C/EBPβ in driving adipose tissue-derived stem cells (ADSCs) and bone marrow-derived MSCs towards hepatic cells; yet, only trivial roles were ascribed to C/EBPα.

(b) Epigenetic modification. Epigenetic modification may contribute to overcome cell fate. determinism of MSCs. As such, we found previously that addition of 1 μM trichostatin A (TSA) to cultured human (h) MSCs, pretreated for 6 days with hepatogenic stimulating agents, triggers their “transdifferentiation” into cells with phenotypic and functional characteristics similar to those of primary hepatocytes [84]. In line with our results, Seo et al. [79] showed enhanced hepatic differentiation upon addition of 0.1% dimethylsulfoxide (DMSO) to hADSCs, prestimulated for 10 days with a mixture of hepatogenic cytokines. Recently, DNA methyl transferase inhibitors (DNMTis), either alone or combined with HDACis, also were introduced to alter cell fate [85–88]. Basically, DNMTis function as preconditioning agents before hepatic differentiation [87–89], whereas HDACis act as stimulants during or after differentiation [68, 79, 90, 91]. In general, chromatin remodeling seems, thus, to be a potential innovative strategy to overcome cell fate determinism and favor lineage-specific differentiation. This field is expected to emerge in the coming years.

### Bipotent Liver Progenitor Cells

LPCs mainly comprise a bipotent progenitor cell population within the liver [92]. Their biliary/hepatic cell fate highly depends on cooperative cross-talks between extrinsic and intrinsic signaling pathways. Soluble factors, in particular, may execute pleiotropic effects (Table 3).

#### Addition of Soluble Medium Factors (Growth Factors/Cytokines/Corticosteroids/Hormones)

Differentiation of LPCs into either the biliary or hepatic lineage greatly depends on the type of growth factor/cytokine used. (a) In midphase fetal liver, transforming growth factor (TGF) β promotes LPCs to undergo biliary differentiation [93], whereas HGF, FMS-like tyrosine kinase 3, stem cell factor (SCF), epidermal growth factor (EGF) [93–96], and members of the Gp130 receptor family, including OSM [97, 98], promote their initial hepatic differentiation and maturation, respectively. FGF propagates embryonic liver cultures toward hepatic progenitors. In this sense, FGF1 and FGF4 enrich for bipotential hepatic progenitors, whereas FGF8 further promotes the former enrichment for unipotential hepatocyte progenitors [99]. (b) In neonatal and adult rodent liver, HGF, FGF (FGF-1, FGF-2, and FGF-4), EGF, SCF, and TGFα,β might simultaneously play a central role in activation/proliferation, maintenance, and differentiation of LPCs such as liver epithelial cells and oval cells [100–103]. Some exceptions do occur, however [104].

Guidance of their cell fate by corticosteroids and hormones is less unidirectional. For example, dexamethasone upregulates the number of both hepatic- and bile duct-like cells in LPCs derived from midphase fetal mouse liver tissue [93]. Despite this scattered effect, when dexamethasone is accompanied by sodium butyrate, cultured oval cells shift solely toward the hepatocyte lineage [105]. Furthermore, growth-promoting effects have been ascribed to insulin, transferrin, α-tocopherol acetate, selenite, linoleic acid, nicotinamide, and hydrocortisone [95, 100–102, 106].

#### Reconstruction of In Vivo Cell-Matrix and Cell-Cell Interactions

The decisive factor of the microenvironment in directing the liver ontogeny underlines the importance of local cell and tissue paracrine signaling. In the context of this rationale, cocultivation of LPCs with stellate cells and mesenchymal feeder layers, including embryonic chick lung mesenchyme, growth-inhibiting embryonic STO fibroblast, or mesenchymal NIH3T3 fibroblast feeder layers, stimulate differentiation along the hepatic lineage [97, 101, 103, 104]. Cultivation in a three-dimensional collagen gel I matrix culture system provides further support [104]. In turn, removal of feeder layers and introduction of Matrigel leads to the formation of bile structures [104].

Besides signals secreted by the surrounding environment, cell density may also trigger differentiation. In essence, the differentiation efficiency is linearly related to the level of confluence [107, 108].

#### Determination of Cell Fate via Epigenetic Modification

Long before their first introduction as cell fate modulators in ES and MSC cultures, epigenetic modification was found to actively contribute to the regulation of liver stem cell responses. The most commonly used HDACis in LPC cultures are DMSO and sodium butyrate [104–106, 108–111]. Depending on the cell type involved and the developmental/differentiation stage of the cells, these HDACis differentially direct cell fate determination. The following classes of HDACis can be distinguished on the basis of their potency to stimulate biliary and/or hepatic differentiation [112]: (a) hepatic-stimulatory sodium butyrate in FNRL cells [110] and WB-F344 cells [109]; (b) biliary- and hepatic-inducing DMSO in HepaRG [113]; and (c) hepatic-stimulatory DMSO and biliary-inducing sodium butyrate in explants of mouse E9.5 liver diverticulum [93] and in primary cultures of rat E12 liver cells [97]. Recently, priming of liver MSCs with the DNMTi 5-azacitidine was also found to trigger functional hepatic differentiation [114].

## FROM STEM CELLS TO HEPATOCYTES: CURRENT CHARACTERIZATION STRATEGIES AND THEIR LIMITATIONS

Stem cell-derived hepatocyte-like cells may be characterized in vitro at four levels: morphological, RNA, protein, and activity levels. Usually, the analytical work is limited to the elucidation of (a) endodermal/hepatogenic RNA transcripts via (quantitative) reverse transcriptase-polymerase chain reaction and (b) proteins by immunofluorescence. The most studied endodermal markers include LETFs (HNF1α,β, HNF3β, HNF4α, and C/EBPα,β), plasma proteins (AFP, ALB, transthyretin [TTR]), and cytoskeletal proteins (CK18, CK8) (Table 1–3). A minority of studies have examined the expression of CYPs and other “late” enzymes such as tryptophan 2,3-dioxygenase (TO) and tyrosine amino transferase (TAT).

The following three features inherent to hepatic stem cell transitions need to be taken into account to perform accurate phenotyping. (a) The differentiation of stem/progenitor cells toward the hepatocyte lineage often involves uncontrolled processes, resulting in a heterogeneous cell population. Genes such as TAT [115], phosphoenolpyruvate carboxykinase [116], and LETFs [117–120] are also expressed in other somatic cells such as lung, intestine, pancreas, and kidney and thus cannot be considered as “true” hepatocyte markers. In addition, genes such as AFP and TTR are both expressed in liver tissue and in the extraembryonic yolk sac [121, 122]. Hence, exclusive analysis of one of the latter markers cannot count as proof for a genuine hepatic phenotype. The need thus arises to identify genes that are predominantly expressed in the liver and not in other tissues, enabling an accurate follow-up of the differentiation process and precise characterization of the end populations. In mouse, CYP7A1 is solely expressed in the liver and not in the yolk sac tissue, fulfilling its function as a reliable hepatocyte marker [36]. Alternatively, the synthesis of urea is a privileged function of hepatocytes [123, 124].

(b) The differentiation of hepatoblasts into hepatocytes is a steady process. It is known that embryonic, fetal, and adult hepatocytes differ in their molecular phenotype [1, 2]. Basically, hepatogenesis in vivo implies serial expression of early (HNF3β, AFP, and TTR), midlate (HNF1α, HNF4α, ALB, and CK18), and late (TO, TAT, C/EBPα, and CYPs) markers [28, 30–32, 125, 126]. Yet, some genes such as TTR and ALB are first expressed in early-midlate embryos and maintain expression in fetal and adult hepatocytes [126]. Positive expression of these genes may not enlighten the present differentiation state properly. AFP, on the other hand, is expressed very early in embryonic development and during the fetal stages. Its expression gradually levels off with increasing development and disappears entirely in adult life [125]. AFP thus represents a reliable marker to discriminate between distinct developmental stages. Alternatively, most, but not all, metabolic and detoxifying enzymes do not become functional before birth. Indeed, during the terminal step of liver organogenesis, the liver becomes a functional, metabolic organ: hepatocytes start to both control the levels of metabolites and serum proteins in the bloodstream and express numerous new genes and proteins related to specific functions of the adult liver [32, 123, 124]. Therefore, to state the differentiation stage of the resultant hepatocyte-like cells, functional assays for enzymes need to be carried out. At present, functional analysis is particularly focused on glycogen uptake, urea metabolism, and ALB secretion. Only a little attention has been paid to other metabolic functions, including CYP450-dependent activity and responsiveness to prototype inducers such as phenobarbital (human CYP2B6 and CYP3A4 and rat CYP2B1/2), rifampicin (CYP3A4), and 3-methylcholantrene (human and rat CYP1A1/2). If one bears in mind that inducible P450-dependent activity is considered to be a key determinant of the functional hepatic phenotype [123, 127], characterization must encompass the above-mentioned metabolic functionality assays as well.

(c) The ultimate proof of functional hepatic behavior is no doubt in vivo transplantation of ex vivo generated stem cells-based hepatic cells in (immunodeficient) animal models with liver injury [58, 70, 79, 82, 83, 87, 128]. Examples of recipients permissive for engraftment of both allogeneic and xenogeneic cells are partially hepatectomized *Pfp*/*Rag2*^−/−^ [82]/nude [79] mice, carbon tetrachloride-injured severe combined immunodeficient (SCID) mice, and urokinase-type plasminogen activator^+/+^/nonobese diabetic-SCID mice [79, 128, 129]. Positive homing, engraftment, repopulation, and functional maturation are basically explored by means of molecular imaging techniques, immunohistochemistry, in situ hybridization, and serology [58, 70, 79, 82, 83, 87, 128–130]. Despite seemingly irrefutable evidence that stem/progenitor cells could contribute to liver reconstitution, caution should be taken with production of false-positive results owing to application of inaccurate labeling techniques [131]. Also, one should keep in mind that, apart from generating fully functional stem cell-derived hepatocytes, other mechanisms including the bystander effect, fusion (cf. Introduction), partial transdifferentiation, and horizontal gene transfer [128, 129] might be responsible. For a more scrupulous insight in this complex matter, we refer the reader to Hengstler et al. [129].

In brief, accurate hepatic phenotyping in vitro should encompass the molecular analysis of a set of (non)specific hepatic markers in combination with ammonia formation and inducible CYP-dependent metabolism as functionality tests. Confirmation of the in vitro obtained results via rigorous in vivo tools might shed light on the therapeutic potential of stem/progenitor cells in various acute and chronic liver disorders.

## STEM CELL TECHNOLOGIES: CURRENT SHORTCOMINGS

### Need for Standardization, Optimization, and Enrichment

ES cells harbor a unique pluripotent versatility compared with other fetal and adult multi- or bipotent stem/progenitor cell populations. They possess the unrestricted capacity to form embryonic and adult cell types, thereby reflecting the distinct developmental stages in vivo. Yet, the use of ES cell/EB technology encounters a complex differentiation environment, lack of organization, and inherent heterogeneity of the system [132–134]. In addition, although EBs may form functional and specialized cell types, including hepatocytes, the differentiation efficiency in number of lineage-specific cell types obtained is rather low [35, 135]. Culture of EBs in the presence of (a) differentiation inducers or (b) biologically derived signals (e.g., conditioned medium or purified growth factors) or other lineage-selective agents have been used to enrich for specific cell populations [133]. Thus, high-purity (70%–80%) ES cell-derived hepatocyte cultures have recently been produced on subculture and fine-tuning of the order/type of cytokine exposure [47, 48]. Yet, spontaneous differentiation is still predominant in many differentiation protocols. Differentiation is thus a default pathway of ES cells rather than replication. The opposite holds for adult stem cells [3]. For this reason, the use of adult stem/progenitor cells is often considered as a potential alternative. Basically, LPCs in culture differentiate either into hepatocytes, bile duct, or both (bipotency) [92, 97, 98, 136, 137]. The choice of matrices is the most important determinant for the direction taken. Lately, evidence has been provided that mesenchymal(-like) stem/progenitor cells from various sources (bone marrow, adipose tissue, skin, placenta, and umbilical cord) could occasionally overcome lineage borders and differentiate into endodermal (hepatocytes) and ectodermal (neural cells) cell types after specific in vitro induction [69, 74–76, 138–140]. It has now become clear that next to identification of hepatogenic cytokines or growth factors, their concentrations, mode of presentation, and order of application [8] also are crucial for hepatic differentiation and subsequent maturation into functional hepatocytes in vitro. As such, sequential exposure of bone marrow MSCs to hepatogenic factors reflecting their secretion pattern during liver embryogenesis in vivo results in a homogeneous population of functional hepatocytes. A downside of adult stem cell technology, however, is the level of reproducibility. Indeed, we found that only 25% of the bone marrow hMSC samples processed were “plastic” and consequently adopted a functional hepatic phenotype (intralaboratory variability). A number of unknown and consequently insufficiently controlled variables could be responsible. For example, the differentiation potential of MSC might depend on the following:

1. The donor. Age, gender, lifestyle (e.g., smoking, alcohol consumption, or drug abuse, health condition (health/disease), intake of pharmaceutical agents, genetic differences, and others [141]. For example, the yield of MSCs within bone marrow is influenced by age, gender, the presence of osteoporosis, and prior exposure to high-dose chemotherapy or radiation [142]. In addition, both the differentiation and self-renewing capacity of bone marrow and liver MSCs was often, although not exclusively [143], found to level off with age [141, 142, 144]. In contrast, the adipogenic and myogenic differentiation ratios of ADSCs are not affected by the donor's age [142]. To date, little is known about the relationship between disease (cancer) and stem cell behavior [142]. Yet, ADSCs derived from patients with gastric cancer were found to retain their endodermal differentiation potential [142, 145].
2. The starting material. The harvest tissue varies, and the original characteristics of the starting material are often poorly defined (e.g., phenotypic profile, heterogeneity/conformity, and passage number). Phenotypic instability and plastic variability are inherent characteristics of MSCs [141, 146–148]. In this context, individual clones of cell lines derived from MSCs have different potentials for differentiation, indicating different stages of determination and levels of plasticity. Physiological alterations, resulting from exposure of clonal MSCs to a specific microenvironment during both proliferation and differentiation, may induce heritable and epigenetic cellular preconditioning, altering their original phenotype and manipulating their predestined cell fate [147]. In this regard, it was previously shown by DiGirolamo et al. [147] that some of the clonally derived MSCs from a single mother colony, expanded in separate cultures and subjected to identical osteogenic conditions, could efficiently differentiate into osteoblasts whereas others could not. This study clearly illustrates that clonal daughter cells, even when derived from a single mother cell, may have a different(ial) potential in response to soluble factors. The ambiguous definition of starting cell material remains a key obstacle for in vitro purposes and might even explain the global nonreproducibility or discrepancies in inter- and intralaboratory results reported thus far.
3. The technology used. From an extensive review of the current literature, it appears that great variety exists among strategies to isolate, purify, expand, and differentiate postnatal stem cells. MSCs lack well-defined characterization and common surface markers that allow accurate isolation via fluorescence-activated cell sorting (FACS). For this reason, bone marrow MSCs are usually, but not exclusively, isolated via the plastic adherence technique. A major drawback of this strategy is its heterogeneous outcome, yielding a phenotypically mixed fibroblastoid cell population [141, 146, 148–150], often contaminated with hematopoietic cells at low passages [147, 151]. Basically, heterogeneity of initial populations hinders interpretation and reciprocal comparison of results among different research groups. Also, molecular cues necessary to enforce in vitro differentiation are complex and therefore are not easily identifiable or reproducible [152].
4. Stress. Architectural and phenotypic diversification in response to stress might be misinterpreted as a true transdifferentiation phenomena. In fact, stem/progenitor cells removed from their natural niche and subsequently grown in a chemical ex vivo environment emit intrinsic (cellular) and chemical stress signals that in turn could lead to cytoskeletal collapse or pseudo-alchemical transitions [25, 26]. Unraveling the mechanisms underlying current successful and failed occurrences of adult stem cell plasticity and transdifferentiation is a complex and speculative undertaking that goes far beyond the scope of this review. Yet, we emphasize caution in interpreting data as spontaneous transitional processes.

It is conceivable that the factors enumerated above are only in part responsible for the variation in results observed in our studies and those of others. In this regard, it was postulated that phenotypic heterogeneity is intrinsic to stem cells because of their asymmetric self-renewal/differentiation potential.

Another critical factor for the commercial and clinical application (potential) of adult stem cells is the development of high-throughput scaling procedures. Today, most strategies to control and manipulate the cellular microenvironment of undifferentiated stem cells and their differentiated progeny are optimized on a laboratory scale. To be of pharmaceutical relevance, miniaturization and scaling up toward industrial needs are obligatory. In this context, bone marrow as source of hMSCs might not be ideal. Indeed, traditional bone marrow procurement procedures are risky for the patient and, in addition, bone marrow is also not readily available and yields only low numbers of multipotent stem cells upon processing [153]. A more easily accessible and readily available source of MSCs is human adipose tissue [75, 77, 78, 154] or human skin. These sources have the additional advantage that they may be obtained from healthy volunteers of diverse ages and gender. For these reasons, the latter alternative MSC sources are currently being explored.

### Epigenetic Modification under Discussion?

Another point of interest is the role of epigenetic modifiers, particularly HDACis, in mediating hepatic-conditioned postnatal progenitor cells toward fully functional hepatocytes. In general, epigenetic modifiers affect a broad variety of cellular processes, including cell cycling, differentiation, and apoptosis [155–158]. For example, previous findings in our laboratory indicated that epigenetic alterations may represent a valuable approach to develop phenotypically stable primary hepatocyte cultures. It was revealed that addition of TSA to isolated primary hepatocytes impedes G_0_/G_1_ cell cycle transition and consequently favors the maintenance of hepatocellular functionality in vitro [155, 156, 158, 159]. Given this principle and the fact that covalent histone modification is central in processes determining lineage-specific gene expression and cell fate decisions [160, 161], we exposed postnatal bone marrow MSCs to TSA to obtain well-functioning mature hepatocytes. Critical factors in this process are onset of exposure, dose, and environmental conditions (cell-cell contact and cell densities) [155, 157, 158] as discussed in the following.

- Timing. Timing seems most essential in transdifferentiation processes. In this regard, addition of 1 μM TSA to undifferentiated bone marrow hMSCs and 0- to 5-day preconditioned bone marrow hMSCs resulted in massive cell death. On the other hand, hMSCs prestimulated with hepatogenic factors for at least 6 days before addition of 1 μM TSA underwent successful hepatic differentiation. Similar results were found by Seo et al. [79]. The importance of timing is also supported by the significant number of failed transdifferentiation experiments, producing nonhepatocyte-like cells [162–165]. In some cases, failure could be ascribed to inaccurately timing of exposure and determined concentrations.
- Dosage. Determination of the HDACi concentration that induces cell cycle arrest is another crucial factor, as the latter is generally a prerequisite for differentiation in vitro [166]. In primary hepatocyte cultures, differentiation and proliferation exclude each other [167]. Concentrations higher than this critical value may result in massive cell death. In preliminary experiments on bone marrow hMSCs, 5–25 μM TSA was found to be cytotoxic whereas 1 μM TSA, added from the 6th day of differentiation on, supported long-term culture and suppression of proliferation. Yet, at the molecular level a rather high apoptotic level was revealed. It was thought to be conceivable that TSA under hepatic-stimulating conditions selectively induced apoptosis of non(hepatic) differentiating cells, and simultaneously promoted the survival of hepatic differentiating cells. Although this is just a hypothesis and thus not based on stated evidence, it does stress the importance of timing and dose optimization of HDACis.
- Biotransformation. TSA is metabolically instable and undergoes intensive phase I biotransformation in primary rat hepatocytes [168]. With a 30-min incubation time, virtually all TSA is metabolized into inactive metabolites. It might thus be optional to use HDACi compounds that are more metabolically stable than TSA [169]; however, the latter only becomes an issue when stem cell-derived hepatocytes acquire metabolic activity.

Another point that can be raised here is the fact that HDACis, being modulators of chromatin, are by nature considered to be genotoxic. To date, data available in the literature are scarce. However, the genotoxic factor may have important consequences once one aims to use HDACi/DNMTi-treated hepatocytes in cell therapy or transplantation.

## PERSPECTIVES

From the discussion in From Stem Cells to Hepatocytes: Current in Vitro Differentiation Strategies and From Stem Cells to Hepatocytes: Current Characterization Strategies and Their Limitations, it becomes clear that standardization of the production of functional hepatocytes out of postnatal progenitors and improvement of the hepatic potency of the initial progenitor population are tasks for the future. Here we state some ideas that may help to guide future stem cell research.

- A precise characterization of the undifferentiated initial cell populations is of utmost importance for future exploitation of stem cell technology. Phenotyping based on surface markers has thus far been insufficient. Instead, characterization should be performed at morphological, molecular, and functional levels. However, if the hypothesis that heterogeneity is inherent to stem cells is true, efforts hereto may be futile. With microarray analysis of gene expression pattern(s) and proteomics we will learn more. It also remains to be clarified whether physiological markers of MSCs and LPCs become lost or undergo changes during isolation and expansion/subculture procedures. Aging and stress during growth and subculture might also affect the phenotype of progenitors [141, 170–173]. Selection of reliable cell surface markers is therefore desirable to accurately isolate, select, and purify well-defined populations of plastic progenitors via FACS. Public accessibility of phenotypic profiling via databases and the Web may facilitate standardization and comparative inter- and intralaboratory studies.
- Stem cells differ significantly in their surface receptor expression profiles for cytokines/growth factors at successive developmental stages [8,27–29,31–33]. Dosage, timing, and combinations of cytokines/growth factors should thus be fine-tuned according to the differentiated state and type of stem cell involved. The suitability of epigenetics to promote hepatic (trans)differentiation requires a delicate balance between biological activity, pharmacokinetic, and toxicological characteristics; proliferation/differentiation; and finally apoptosis/cell survival. Successful improvement of the hepatocellular phenotype and functionality of stem cell cultures relies, as is the case for primary hepatocyte cultures, on appropriate selection of type of epigenetic modifier applied and optimal fine-tuning of its dose and timing of exposure [163].
- Another major consideration is the dichotomy between hepatocyte proliferation and expression of differentiated functions (overview in [166]). In contrast to the in vivo situation, in which cellular proliferation and differentiation go hand in hand, in vitro differentiation is mostly associated with cell cycle arrest (with the exception of some in vitro cultured LPCs) [102, 103]. Most commonly, cells exit from the cell cycle and then undergo differentiation, resulting in either a terminal, irreversible cell specialization or a particular developmental step in the life cycle [166]. Hence, the dosage and combination of soluble medium additives should be fine-tuned, according to this dichotomy between proliferation and differentiation of the cells.
- Finally, in addition to variability at the in vitro level, it should be clarified whether or not the multipotency of stem/progenitor cells significantly depends on the donor's profile [141, 171, 174]. Simple questions on the effect of age (young or elderly donors), lifestyle (e.g., smokers or nonsmokers), health condition, and other factors should be answered before practical application is considered.

In conclusion, a more scrupulous understanding of the instructive signals emanating from the stem cell niche, together with a deeper analysis of cell-intrinsic mechanisms governing replication versus differentiation-inducing signals, is needed to reliably expand and differentiate stem/progenitor cells. Identification of reliable surface markers, useful for accurate and efficient isolation of plastic progenitor cells may upregulate the current hepatic potential of MSC and eventually serve to construct efficient and standardized devices that enable specific direction of MSCs and other progenitors towards the hepatocyte lineage. Standardization is, in any case, a sine qua non for prospective preclinical and clinical purposes of stem cells and their differentiated progeny.
